# The relationship between frequency and severity of vaso-occlusive crises and health-related quality of life and work productivity in adults with sickle cell disease

**DOI:** 10.1007/s11136-019-02412-5

**Published:** 2020-01-13

**Authors:** Avery A. Rizio, Menaka Bhor, Xiaochen Lin, Kristen L. McCausland, Michelle K. White, Jincy Paulose, Savita Nandal, Rashid I. Halloway, Lanetta Bronté-Hall

**Affiliations:** 1 Patient Insights, Optum, 1301 Atwood Ave, Suite 311N, Johnston, RI USA; 2 Novartis Pharmaceutical Corporation, One Health Plaza, East Hanover, NJ USA; 3 Formerly Novartis Pharmaceutical Corporation, One Health Plaza, East Hanover, NJ USA; 4 Foundation for Sickle Cell Disease Research, 3858 Sheridan St, Suite S, Hollywood, FL USA

**Keywords:** Sickle cell disease, Vaso-occlusive crises, Health-related quality of life, Work productivity

## Abstract

**Purpose:**

Patients with sickle cell disease (SCD) may experience sickle cell-related pain crises, also referred to as vaso-occlusive crises (VOCs), which are a substantial cause of morbidity and mortality. The study explored how VOC frequency and severity impacts health-related quality of life (HRQoL) and work productivity.

**Methods:**

Three hundred and three adults with SCD who completed an online survey were included in the analysis. Patients answered questions regarding their experience with SCD and VOCs, and completed the Adult Sickle Cell Quality of Life Measurement Information System (ASCQ-Me) and the Workplace Productivity and Activity Impairment: Specific Health Problem (WPAI:SHP). Differences in ASCQ-Me and WPAI:SHP domains were assessed according to VOC frequency and severity.

**Results:**

Nearly half of the patient sample (47.2%) experienced ≥ 4 VOCs in the past 12 months. The most commonly reported barriers to receiving care for SCD included discrimination by or trouble trusting healthcare professionals (39.6%, 33.3%, respectively), limited access to treatment centers (38.9%), and difficulty affording services (29.4%). Patients with more frequent VOCs reported greater impacts on emotion, social functioning, stiffness, sleep and pain, and greater absenteeism, overall productivity loss, and activity impairment than patients with less frequent VOCs (*P* < 0.05). Significant impacts on HRQoL and work productivity were also observed when stratifying by VOC severity (*P* < 0.05 for all ASCQ-Me and WPAI domains, except for presenteeism).

**Conclusions:**

Results from the survey indicated that patients with SCD who had more frequent or severe VOCs experienced deficits in multiple domains of HRQoL and work productivity. Future research should examine the longitudinal relationship between these outcomes.

## Introduction

Sickle cell disease (SCD) is a hemoglobinopathy that causes red blood cells to lose their oxygen carrying capacity and is associated with severe, systemic vascular complications. It is estimated that approximately 100,000 Americans have SCD [1]. Patients with SCD experience chronic pain, cardiovascular events, ulcers, fatigue, organ damage, and sickle cell-related pain crises, also referred to as vaso-occlusive crises (VOCs). Treatments used to manage symptoms or reduce complications of SCD include hydroxyurea, l-glutamine, and blood transfusions [2]; currently, the only available cure for SCD is bone marrow transplant [3].

Previous research has provided insight into many of the ways in which patients are impacted by SCD [4]. For example, patients with SCD report experiencing sleep disturbances [5], as well as deficits in both physical and mental well-being [6]. The extensive burden of SCD may also lead to an inability to maintain consistent work or schooling, engage in daily, social, or recreational activities, and participate in family life [7–10]. In addition to the burden of SCD, the experience of VOCs also has detrimental impacts on the lives of patients, though these impacts have been less comprehensively studied. VOCs are caused by multi-cell adhesion or cell clusters that block or reduce blood flow, and are a substantial cause of morbidity in patients with SCD; severe crises have also been associated with increased mortality [11]. These events are unpredictable and can cause disruption and hardship in the lives of patients, sometimes requiring medical attention in emergency departments or sickle cell urgent care centers, or leading to inpatient hospitalization [12]. Previous occurrence of VOCs has been linked to deficits in domains of health-related quality of life (HRQoL) such as general health, vitality, and bodily pain [6].

SCD is associated with high healthcare resource utilization (HCRU), with VOCs being the most common cause of hospital and emergency department visits among patients with SCD [13]. High rates of HCRU have been linked to a variety of poor outcomes among patients with SCD, including lower HRQoL and likelihood of unemployment [14–17]. Despite high HCRU, especially for acute treatment of VOCs [18], the type of care patients with SCD receive may nevertheless be suboptimal. Receiving adequate treatment is a challenge commonly faced by patients with SCD, particularly for those who are transitioning from pediatric to adult care. Patients may have difficulty finding experienced practitioners to treat their SCD, coordinating communication between providers in a multi-disciplinary care setting, and obtaining or maintaining adequate insurance coverage, all of which could in turn result in over-reliance on emergency departments for treatment [19]. Perhaps unsurprisingly, patients themselves indicate that they prefer treatments they can administer at home [20], and report managing most of their VOCs at home [21]. Research has suggested that patients who prefer treating their pain at home believe that going to a hospital is not in their best interest and feel that they are responsible for managing their pain [22].

Given the seriousness of VOCs, there is an on-going need to better understand the ways in which these events impact patients with SCD. Although patients themselves have identified VOCs as one of the most debilitating aspects of their disease [20], minimal research has been conducted to quantify the extent of their impact on patients’ lives. Therefore, additional research into their effect on patients’ HRQoL and other outcomes is needed to provide insight into areas of unmet need and guide treatment development. Recognizing these gaps in the literature, the goal of this study was to examine the relationship between the frequency and severity of VOCs and HRQoL and work productivity impairment, using patient-reported data.

## Methods

### Sample/study procedures

The data for this analysis were drawn from an online, non-interventional, and cross-sectional study of adults with SCD. The study was approved by the New England Independent Review Board, and informed consent was obtained from all participants.

The patients were invited to participate in the study in 2018 through collaboration with patient advocacy groups and a market research company (Schlesinger Group). These groups distributed a description of the study, along with a hyperlink to the study’s screening page, to potential participants. Patients who followed the hyperlink were directed to complete the screening survey. Patients were eligible to participate if they were aged ≥ 18 years, self-reported having been diagnosed with SCD by a physician, currently resided in the US, and were willing to complete the online survey in English. Those who were deemed eligible to participate were automatically directed to complete the informed consent form, followed by the survey. Patients received the equivalent of a $75 gift card for completing the survey. Data collection began on November 9, 2018, and ended on January 22, 2019.

### Study measures

The online survey consisted of multiple modules designed to assess a variety of aspects of the patient experience, including demographic and disease characteristics; HRQoL; work productivity; VOC-related treatment experiences, HCRU, and management; barriers to receiving care; and impacts of SCD on employment, education, and personal relationships.

#### Health-related quality of life

The Adult Sickle Cell Quality of Life Measurement Information System (ASCQ-Me) is a disease-specific measure of HRQoL for patients with SCD [23]. The overall measurement system assesses 7 different health topics; 6 of these topics are assessed through 5-item questionnaires (Emotional Impact, Pain Impact, Sleep Impact, Social Functioning Impact, Stiffness Impact, Pain Episode), while the seventh topic is assessed through a 9-item questionnaire (SCD Medical History Checklist). The Pain Episode domain assesses the frequency of VOCs experienced in the past 12 months as well as the severity and degree of impact of the most recent VOC. Each of the 5 items in the Pain Episode domain can be used individually to describe the patient sample, or can be used together to create 2 composite scores: Pain Episode Frequency and Pain Episode Severity. This study used the static electronic forms to assess all domains except the SCD Medical History Checklist.

Each of the forms was scored according to developer guidelines and transformed to *t* scores [23]. *T* scores are standardized to have a mean of 50 and a standard deviation (SD) of 10, where a score of 50 represents the average SCD patient’s HRQoL from a benchmark population of adults with SCD [23]. Higher domain scores represent a more favorable status for the Emotional, Pain, Sleep, Social Functioning, and Stiffness Impacts domains. Lower domain scores represent a more favorable status for the Pain Episode Frequency and Pain Episode Severity scores. The ASCQ-Me uses the phrase “pain attacks (crises)” to refer to VOCs and thus was adopted throughout the entirety of the online survey. This language was assessed during the original validation of the instrument, where it was determined that patients generally interpreted the phrase as the developers intended [23–26].

#### Work productivity

The Work Productivity and Activity Impairment: Specific Health Problem (WPAI:SHP) is a 6-item, self-report measure that assesses the impact of a person’s specific health problem on work and daily non-work-related activities during the preceding week [27]. For this study, patients responded to each question in reference to their SCD.

The WPAI:SHP is scored to yield 4 domain scores. Amount of work time missed (absenteeism), impairment while at work (presenteeism), and overall productivity loss (absenteeism and presenteeism combined) are calculated for currently employed patients only. Activity impairment is calculated for all patients, regardless of current employment status, and reflects impairment in daily activities due to SCD. All WPAI:SHP domain scores are expressed as percentages, where larger values indicate greater impairment.

#### VOC-related treatment experiences, healthcare resource utilization, and management

Patients were presented with several questions related to treatment experiences and management of VOCs, including self-reported HCRU. Patients were asked to report the number of healthcare provider visits (not including visits to a hospital emergency room (ER), urgent care, or inpatient admission), the number of hospital ER or urgent care visits, and the number of hospital admissions they had in the past 12 months for treatment of VOCs. Patients were also asked to report where they typically receive treatment for VOCs. Those who reported managing at least 1 VOC at home in the past 12 months were asked to report on the types of treatment they use and the reason they chose to treat their VOCs at home.

#### Barriers to receiving treatment, and SCD-related impacts on employment, education, and personal relationships

Patients were asked whether they had experienced any barriers to receiving SCD-related healthcare services. Response options were informed through literature review, a patient advisory board meeting, and clinician review. Patients were also asked to indicate whether SCD had ever impacted their employment status, education, or personal relationships. Patients who indicated that they had experienced negative impacts were asked to select from a list of specific negative outcomes which they had experienced (e.g., employment impacts: lost a job because of SCD, had to reduce work hours because of SCD; education impacts: dropped out of a school program because of SCD, did not enter a post-secondary school program because of SCD). For all barrier and impact-related items, patients could select more than one response option.

### Statistical analyses

Descriptive statistics, such as frequencies and proportions (for categorical variables) and means, SDs, medians, and ranges (for continuous variables), were used to describe the sample in terms of patient characteristics, HRQoL scores, and WPAI scores. Descriptive statistics were also used to describe patients’ treatment experiences and management of VOCs.

To examine the association between VOC frequency (or severity) and outcomes related to HRQoL and work impairment, a series of bivariate analyses were conducted.

Before the analyses were conducted, patients were stratified based on VOC frequency and severity. To assess VOC frequency, the first item of the ASCQ-Me Pain Episode domain was used; this item asks patients to report the number of VOCs they experienced in the past 12 months. Patients were stratified into one of 2 groups: those who experienced 0–3 VOCs in the past 12 months and those who experienced ≥ 4 VOCs in the past 12 months. This stratification was based on the distribution of data. To assess VOC severity, the Pain Episode Severity score of the ASCQ-Me was used. The items that comprise this score assess severity of pain during the patient’s last VOC, the degree to which the last VOC interfered with the patient’s life, and the duration of the patient’s last VOC. As previously described, the ASCQ-Me domains are scored relative to an SCD benchmark mean of 50 and an SD of 10, with higher Pain Episode scores indicating less favorable status. Because there are currently no guidelines regarding what constitutes a “severe” or “less severe” VOC, the distribution of SCD benchmark scores was used to inform patient stratification. Specifically, patients whose ASCQ-Me Pain Episode Severity score was at least ½ SD more severe than the SCD benchmark (i.e., scores ≥ 55) were categorized as having “more severe” VOCs [28]. Patients whose ASCQ-Me Pain Episode Severity score was < 55 were categorized as having “less severe” VOCs.

Kruskal–Wallis tests were conducted to explore the relationship between VOC frequency (or severity) and HRQoL and work productivity outcomes. Emotional, Social Functioning, Sleep, Stiffness, and Pain, as assessed by the ASCQ-Me, were used as the measures of HRQoL. In addition to testing for statistical significance between groups according to VOC frequency (or severity), average scores were compared to the SCD benchmark score of 50. Because no formal minimal importance difference (MID) has been established for the ASCQ-Me, ½ SD of the average benchmark score (5 points), was used as the threshold to determine scores that differed meaningfully from the SCD benchmark score [28]. Absenteeism, presenteeism, overall productivity loss, and activity impairment, as assessed by the WPAI, were used as the measures of work productivity. Significance of all tests was assessed using an alpha level of 0.05.

If a patient was missing data for a certain item, they were excluded from any analysis that used that item. All analyses were performed using SAS software version 9.4 (SAS Institute Inc.; Cary, NC, USA).

## Results

### Analytic sample

**Fig. 1:**
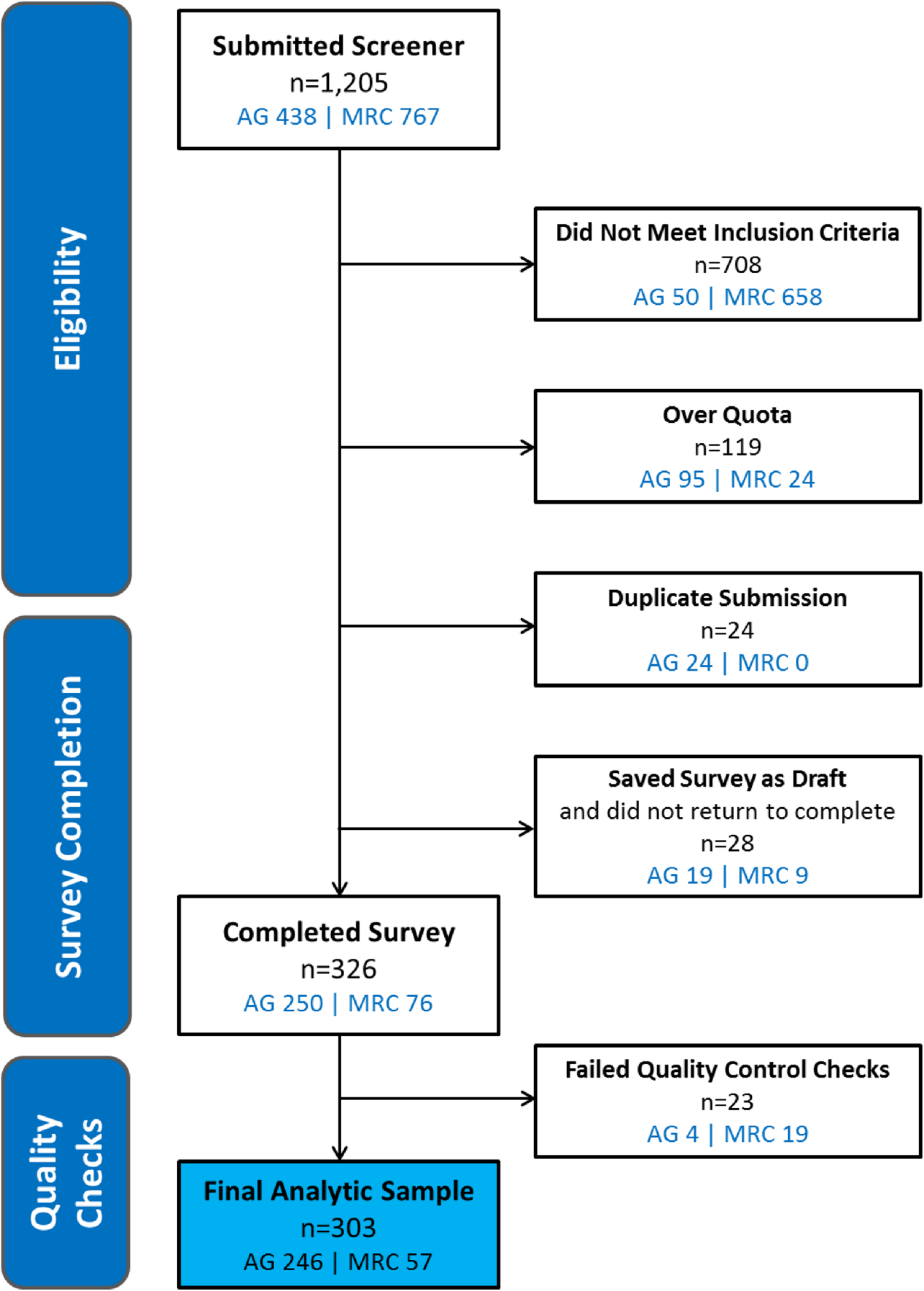
Flowchart of patient disposition. *AG* advocacy groups, *MRC* market research company

Of the 326 patients who completed the survey, 23 reported inconsistent responses across multiple survey items and thus were excluded from analysis. Inconsistent responses were defined either as (1) different responses to 2 items (one administered at screening and one administered later as part of the survey) regarding the number of VOCs experienced in the past 12 months in conjunction with a survey completion time of < 9 min (completion time at or below the 25^th^ percentile among an interim sample of patients) or (2) inconsistent responses to items within the ASCQ-Me Pain Episode domain (e.g., reporting that they experienced 4 or more VOCs in the past 12 months [item 1 of the ASCQ-Me Pain Episode domain], followed by reporting that their last VOC was more than 5 years ago [item 2 of the ASCQ-Me Pain Episode domain]). The final analytic sample included 303 patients (Fig. 1).

### Sample characteristics

The characteristics of the patient sample are depicted in Table 1. The majority of patients were female and black or African American, and there was diversity in the patient sample in terms of educational attainment, type of health insurance, and US region of residence. The most frequently reported type of SCD within the sample was Hb-SS, followed by Hb-SC. Patients reported receiving a variety of treatments for their SCD, including folic acid (*n* = 209, 69.0%), opioid therapy (*n* = 176, 58.1%), NSAIDS (*n* = 125, 41.3%), hydroxyurea (*n* = 119, 39.3%), IV fluids (*n* = 115, 38.0%), and blood transfusions (*n* = 104, 34.3%) (data not shown). Average ASCQ-Me scores were all within ½ SD from the SCD benchmark score of 50, with SDs of 10 or less.

**Table 1:** Patient demographics, clinical characteristics, and health-related quality of life and work impairment scores

Demographic characteristics	Patients with SCD (*N* = 303)	
	*n*	%
Age, mean years (SD), median (IQR)	34.37 (10.25)	33.00 (27.00–40.00)
Gender		
Female	221	72.9
Male	81	26.7
Non-binary/third gender	1	0.3
Race^a^		
Black or African American	270	89.7
White	15	5.0
Asian	2	0.7
American Indian or Alaska Native	1	0.3
Native Hawaiian or other Pacific Islander	1	0.3
Multiple races	6	2.0
Prefer not to answer	6	2.0
Education		
Less than high school or some high school	18	6.0
High school or equivalent (e.g., GED)	39	12.9
Some college, technical school, or associate’s degree	136	45.0
4-year college degree (e.g., BA, BS)	67	22.1
Some graduate school but no degree	12	4.0
Graduate or professional degree (e.g., MBA, MS, MD, PhD)	31	10.2
Employment Status^b^		
Currently employed (working for pay)	120	39.7
Unemployed	182	60.3
Health insurance^c^		
Private insurance	106	35.0
Medicaid	134	44.2
Medicare	103	34.0
Veterans Health Insurance	2	0.7
Other (other type of insurance not listed, uninsured, or unsure)	37	12.2
US region of residence^a,d^		
Northeast	34	11.3
South	167	55.5
Midwest	66	21.9
West	34	11.3
Type of SCD^b^		
Hb-SS	156	51.7
Hb-SC	60	19.9
Hb-S beta^+^ thalassemia	20	6.6
Hb-S beta^0^ thalassemia	22	7.3
Other or do not know	44	14.5

Health-related quality of life	Mean (SD)	Median (IQR)
ASCQ-Me^e^		
Emotional impact	46.57 (8.00)	46.20 (41.20–51.50)
Social functioning impact	47.00 (9.21)	47.20 (40.40–52.20)
Stiffness impact	46.54 (8.35)	46.70 (42.70–51.00)
Sleep impact	48.71 (6.54)	48.20 (45.00–53.90)
Pain impact	47.87 (9.48)	47.10 (41.50–54.00)
Pain episode frequency	49.05 (10.65)	51.85 (44.07–55.73)
Pain episode severity	51.11 (10.41)	52.30 (45.28–59.31)

Work impairment	Mean (SD)	Median (IQR)
WPAI:SHP^f^		
Absenteeism	27.54 (29.04)	20.00 (0.00–50.00)
Presenteeism	47.19 (30.50)	50.00 (20.00–70.00)
Overall work productivity loss	55.19 (33.28)	61.51 (28.89–85.00)
Overall activity impairment	53.15 (28.88)	60.00 (30.00–80.00)

*ASCQ-Me* Adult Sickle Cell Quality of Life Measurement Information System; *WPAI:SHP* Work Productivity and Activity Impairment: Specific Health Problem, *SCD* sickle cell disease, *SD* standard deviation, *IQR* inter-quartile range

^a^Data from 2 patients are missing; frequency based on available data (*N* = 301)

^b^Data from 1 patient is missing; frequency based on available data (*N* = 302)

^c^Multiple response options allowed; frequency sums to > 100%

^d^Regions defined according to US Census Bureau

^e^Higher ASCQ-Me impact scores indicate better functioning. Higher ASCQ-Me pain episode scores indicate worse functioning

^f^Higher WPAI:SHP scores indicate greater impairment. Absenteeism scores were calculated for patients who were employed at the time of the survey (*N* = 118). Presenteeism and overall work productivity scores were calculated for patients who were both employed and reported working in the past 7 days (*N* = 114). Overall activity impairment scores calculated for all patients (*N* = 302; data from 1 patient is missing)

### VOC-related treatment experiences, healthcare resource utilization, and management

#### Treatment experiences

As depicted in Table 2, the majority of patients had experienced at least 1 VOC in the past 12 months (*n* = 276, 91.1%); nearly half of the sample (*n* = 143, 47.2%) experienced 4 or more VOCs during this time frame. Patients reported seeking treatment for VOCs at a variety of locations; the most frequently endorsed locations were the ER and at home.

**Table 2:** Treatment experiences and management of  VOCs

	Patients with SCD (*N* = 303)	
	*n*	%
Number of VOCs experienced in the past 12 months		
0	27	8.9
1	29	9.6
2	44	14.5
3	60	19.8
4 or more	143	47.2
Location at which patients typically receive treatment for VOCs^a,b^		
Home	150	51.0
Primary care doctor’s office	54	18.4
Hematologist’s office	108	36.7
Specialized SCD center, acute care center, or day clinic	63	21.4
Hospital outpatient clinic	67	22.8
Hospital inpatient setting	118	40.1
ER or urgent care	207	70.4
Treatments patients use to manage VOCs at home^a,c^		
Non-narcotic analgesics (e.g., Tylenol, aspirin, Advil)	84	61.3
Mild narcotic analgesics/opioids (e.g., codeine, oxycodone)	87	63.5
Strong narcotic analgesics/opioids (e.g., morphine, hydromorphone, meperidine)	61	44.5
Herbal medicines	44	32.1
Homeopathic remedies	28	20.4
Mind/body practices (e.g., meditation, relaxation techniques, yoga)	75	54.7
Other non-drug therapies (e.g., rest, fluids, heating pad)	106	77.4
Other	7	5.1
Reasons patients treat their VOCs at home^a,c^		
I know what to do to treat my pain	105	76.6
My pain is mild	49	35.8
I do not consider the need to go elsewhere to treat my pain attacks (crises)	17	12.4
I have limited or no access to other treatment options	19	13.9
I do not think others are able or willing to treat my pain attacks (crises)	21	15.3
It is difficult to find transportation to receive treatment elsewhere	18	13.1
It is too expensive to receive treatment elsewhere	25	18.2

	Mean (SD)	Median (range)
HCRU for VOCs in the past 12 months		
Number of visits to a health provider for treatment of VOCs^d^	4.78 (6.11)	3.00 (0–48)
Number of visits to a hospital ER and/or urgent care for treatment of VOCs^d^	5.27 (10.34)	3.00 (0–100)
Number of overnight or longer hospital stays for treatment of VOCs^d^	4.17 (8.81)	2.00 (0–100)
Number of nights spent in hospital during each overnight or longer hospital stay for treatment of VOCs^e^	6.73 (10.00)	4.00 (1–100)

*ER* emergency room, *SCD* sickle cell disease, *SD* standard deviation, *VOCs* vaso-occlusive crises, *HCRU* Healthcare resource utilization

^a^Multiple response options allowed; frequency sums to > 100%

^b^Items administered only to patients who indicated they have experienced at least one VOC in their lifetime (*N* = 294)

^c^Item administered only to patients who have managed at least one VOC at home in the past 12 months (*N* = 137)

^d^Item administered only to patients who indicated they have experienced at least one VOC in the past 12 months (*N* = 276)

^e^Item administered only to patients who indicated they were hospitalized overnight for VOCs in the past 12 months (*N* = 215)

#### Home-managed VOCs

Patients who had reported treating at least 1 VOC at home in the past 12 months reported using a variety of treatment methods including non-drug therapies, mind–body practices (such as meditation), non-narcotic analgesics, and mild narcotic analgesics. The majority of patients (*n* = 105, 76.6%) reported that they treat their VOCs at home because they know how to treat their pain (Table 2).

#### Healthcare resource utilization

Average HCRU for VOCs among patients who experienced at least 1 VOC in the past 12 months is also reported in Table 2. While some patients did not report using specific healthcare services for VOCs, the majority of the sample reported at least 1 healthcare visit in the past 12 months.

### Barriers to receiving treatment, and SCD-related impacts on employment, education, and personal relationships

Approximately three-quarters of patients reported experiencing at least 1 barrier to receiving treatment for SCD (Table 3). 59.1% of patients (*n* = 179) reported that SCD had negatively impacted their employment status. Patients most frequently reported that they had to stop working (*n* = 105, 58.7%), take a leave of absence/unpaid time off (*n* = 94, 52.5%), or reduce work hours (*n* = 89, 49.7%) because of SCD.

**Table 3:** Barriers to care and impacts on employment, education, and personal relationships due to SCD

	Patients with SCD (*N* = 303)	
	*n*	%
Barriers patients have experienced to receiving healthcare for SCD^a^		
Difficulty affording healthcare services	89	29.4
Limited or lack of health insurance	80	26.4
Difficulty obtaining transportation to receive healthcare services	51	16.8
Discrimination or stigmatization by healthcare professionals	120	39.6
Difficulty trusting healthcare professionals	101	33.3
Limited or lack of specialized SCD centers, acute care centers, or day clinics	118	38.9
Other	16	5.3
None of the above	74	24.4
Impact on employment^a^		
Yes, it has positively impacted my employment status	31	10.2
Yes, it has negatively impacted my employment status	179	59.1
No, it has not impacted my employment status	76	25.1
Not applicable	25	8.3
Type of negative employment impacts^a,b^		
Lost a job because of SCD	69	38.5
Stopped working because of SCD	105	58.7
Changed jobs/professions because of SCD	54	30.2
Reduced work hours because of SCD	89	49.7
Took a leave of absence or unpaid time off because of SCD	94	52.5
Changed job responsibilities because of SCD	52	29.1
Have not sought a promotion, or been granted a promotion because of SCD	39	21.8
Other	21	11.7
Impact on education^a^		
Yes, it has positively impacted my level of education	48	15.8
Yes, it has negatively impacted my level of education	128	42.2
No, it has not impacted my level of education	107	35.3
I do not know / I am not sure	30	9.9
Type of negative education impacts^a,c^		
Did not enter a post-secondary school program (e.g., college or technical school) because of SCD	9	7.0
Changed area of study because of SCD	29	22.7
Delayed beginning a school program because of SCD	32	25.0
Delayed finishing a school program because of SCD	60	46.9
Dropped out of a school program because of SCD	56	43.8
Other	27	21.1
Negative impact on personal relationships		
Yes	153	50.5
No	106	35.0
I do not know/I am not sure	35	11.6
Not applicable	9	3.0

*SCD* sickle cell disease

^a^Multiple response options allowed; frequency sums to > 100%

^b^Items administered only to patients who indicated experiencing a negative impact on employment (*N* = 179)

^c^Items administered only to patients who indicated experiencing a negative impact on education (*N* = 128)

42.2% of patients (*n* = 128) reported that SCD had negatively impacted their education (Table 3). Patients most frequently reported that they had either delayed finishing a school program (*n* = 60, 46.9%) or dropped out of a school program (*n* = 56, 43.8%) because of SCD. Half of the patients (*n* = 153, 50.5%) reported that SCD had negatively impacted their ability to start or continue a relationship.

### Health-related quality of life according to VOC frequency and severity

**Fig. 2:**
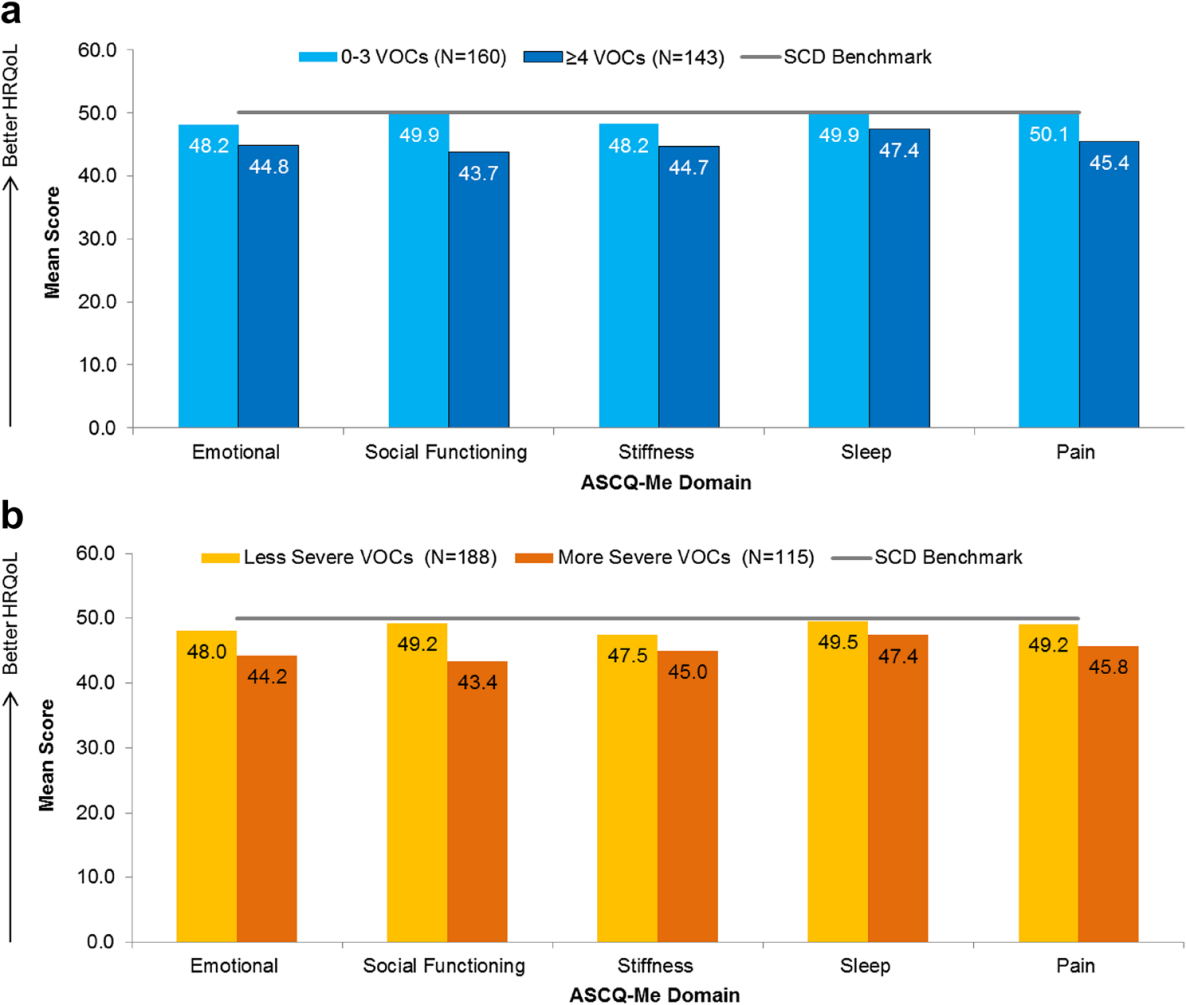
HRQoL according to VOC frequency (**a**) and severity (**b**). HRQoL scores differ as a function of VOC frequency/severity for all domains (*P* < 0.05). Higher ASCQ-Me impact scores indicate better functioning. Less severe VOCs = ASCQ-Me Pain Episode Severity score < 55; more severe VOCs = ASCQ-Me Pain Episode Severity score ≥ 55. *ASCQ-Me* Adult Sickle Cell Quality of Life Measurement Information System, *HRQoL* health-related quality of life, *SCD* sickle cell disease, *VOCs* vaso-occlusive crises

Statistically significant differences in emotion, social functioning, stiffness, sleep, and pain were observed when stratifying patients according to the frequency of their VOCs over the past 12 months (Table 4; Panel a of Fig. 2). Patients with more frequent VOCs reported worse HRQoL across all of these domains, as measured by the ASCQ-Me, than patients with less frequent VOCs. Patients who experienced 0–3 VOCs in the past 12 months reported HRQoL scores that were equivalent to the SCD benchmark score of 50, meaning that their scores were similar to that of the average SCD patient, as defined by the benchmark sample. However, patients who experienced ≥ 4 VOCs in the past 12 months reported emotion, social functioning, and stiffness impacts that were ½ SD worse than the SCD benchmark.

**Table 4:** HRQoL according to VOC frequency

ASCQ-Me domain^a^	0–3 VOCs in the past 12 months					≥ 4 VOCs in the past 12 months					*P*^b^
	*N*	Mean	SD	Median	IQR	*N*	Mean	SD	Median	IQR	
Emotional impact	160	48.16	8.33	47.40	42.50–54.25	143	44.80	7.23	44.90	39.90–48.70	0.001
Social functioning impact	160	49.92	9.48	48.80	43.90–55.80	143	43.73	7.71	43.90	38.70–48.80	< 0.001
Stiffness impact	160	48.20	8.72	48.10	42.70–52.70	143	44.67	7.52	45.40	39.90–49.50	0.001
Sleep impact	160	49.87	6.65	49.70	45.00–55.30	143	47.42	6.19	46.70	43.20–51.10	< 0.001
Pain impact	160	50.06	9.49	48.50	44.40–58.00	143	45.41	8.89	45.70	38.30–51.20	< 0.001

*ASCQ-Me* Adult Sickle Cell Quality of Life Measurement Information System, *VOCs* vaso-occlusive crises, *SD* standard deviation, *IQR* inter-quartile range, *HRQoL* Health-related quality of life

^a^Higher ASCQ-Me impact scores indicate better functioning

^b^After Bonferroni correction, all associations remained statistically significant, with *P* < the Bonferroni correction-adjusted critical value of 0.01

An additional set of analyses was conducted to explore HRQoL according to different stratifications of VOC frequency. Specifically, patients were divided into 3 groups: 0 VOCs, 1 VOC, and ≥ 2 VOCs. The overall pattern of results remained unchanged, as patients with ≥ 2 VOCs in the past 12 months had lower (i.e.,worse) HRQoL scores than patients with 0 or 1 VOCs (data not shown). Due to the limited number of patients who experienced either 0 or 1 VOC in the past 12 months, however, this stratification was not pursued for the other outcomes of interest (i.e., work impairment).

Statistically significant differences in emotion, social functioning, stiffness, sleep, and pain were also observed when stratifying patients according to the severity of their last VOC (Table 5; Panel b of Fig. 2). Patients with less severe VOCs reported mean scores across all domains that were similar to the SCD benchmark score; patients with more severe VOCs reported mean emotion, social functioning, and stiffness scores that were ½ SD worse than the SCD benchmark.

**Table 5:** HRQoL according to VOC severity

ASCQ-Me domain^a^	Less severe VOCs					More severe VOCs					*P*^b^
	*N*	Mean	SD	Median	IQR	*N*	Mean	SD	Median	IQR	
Emotional impact	188	48.00	7.36	47.40	43.10–51.50	115	44.24	8.48	43.70	39.90–48.70	< 0.001
Social functioning impact	188	49.22	8.61	48.80	43.90–54.00	115	43.37	9.05	42.10	36.80–47.20	< 0.001
Stiffness impact	188	47.49	7.80	46.70	44.00–51.00	115	44.97	8.99	45.40	38.40–49.50	0.008
Sleep impact	188	49.52	6.58	49.70	45.00–53.90	115	47.40	6.29	46.70	43.20–51.10	0.005
Pain impact	188	49.16	8.93	47.10	44.40–55.80	115	45.75	10.00	44.40	38.30–51.20	0.002

More severe VOCs: ASCQ-Me Pain Episode Severity score ≥ 55 (½ SD worse than the benchmark score); less severe VOCs: ASCQ-Me Pain Episode Severity score < 55

*ASCQ-Me* Adult Sickle Cell Quality of Life Measurement Information System, *VOCs* vaso-occlusive crises, *SD* standard deviation, *IQR* inter-quartile range, *HRQoL* Health-related quality of life

^a^Higher ASCQ-Me impact scores indicate better functioning

^b^After Bonferroni correction, all associations remained statistically significant, with *P* < the Bonferroni correction-adjusted critical value of 0.01

### Work productivity according to VOC frequency and severity

**Fig. 3:**
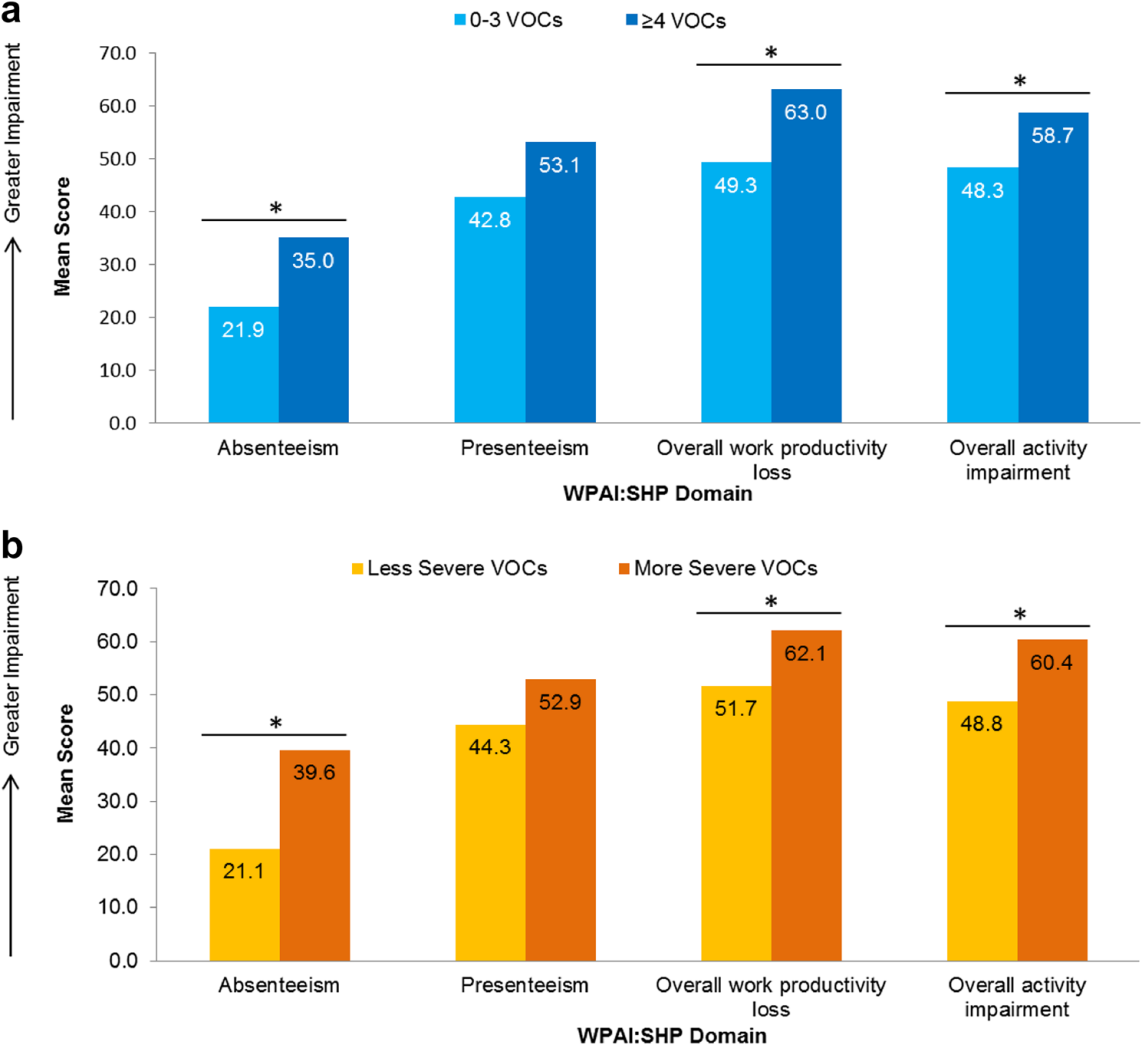
Work impairment according to VOC frequency (**a**) and severity (**b**). *WPAI:SHP domain scores differed as a function VOC frequency/severity (*P* < 0.05). Higher WPAI:SHP scores indicate greater impairment. For sample sizes for per VOC category and WPAI:SHP domain, refer to Tables 6 and 7. Less severe VOCs = ASCQ-Me Pain Episode Severity score < 55; more severe VOCs = ASCQ-Me Pain Episode Severity score ≥ 55. *WPAI:SHP* Work Productivity and Activity Impairment: Specific Health Problem, *VOCs* vaso-occlusive crises

Statistically significant differences in 3 of the 4 WPAI scores were observed when patients were stratified by VOC frequency (Table 6; Panel a of Fig. 3). Specifically, patients with ≥ 4 VOCs in the past 12 months reported greater absenteeism, overall work productivity loss, and activity loss than patients with 0–3 VOCs (*P* < 0.05 for all); presenteeism did not differ significantly according to VOC frequency.

**Table 6:** Work productivity according to VOC frequency

WPAI:SHP domain^a^	0–3 VOCs in the past 12 months					≥ 4 VOCs in the past 12 months					*P*^b^
	*N*	Mean	SD	Median	IQR	*N*	Mean	SD	Median	IQR	
Absenteeism	67	21.87	26.25	14.29	0.00–36.84	51	34.99	31.06	30.77	0.00–57.14	0.018
Presenteeism	65	42.77	30.49	50.00	10.00–70.00	49	53.06	29.81	60.00	30.00–80.00	0.068
Overall work productivity loss	65	49.27	34.01	55.00	10.00–81.05	49	63.03	30.90	70.00	30.00–88.89	0.016
Overall activity impairment	160	48.25	29.96	50.00	20.00–70.00	142	58.66	26.66	60.00	40.00–80.00	0.003

*WPAI:SHP* Work Productivity and Activity Impairment: Specific Health Problem, *VOCs* vaso-occlusive crises, *SD* standard deviation, *IQR* inter-quartile range

^a^Higher WPAI:SHP scores indicate greater impairment. Absenteeism scores were calculated for patients who were employed at the time of the survey (*N* = 118). Presenteeism and overall work productivity scores were calculated for patients who were both employed and reported working in the past 7 (*N* = 114). Overall activity impairment scores calculated for all patients (*N* = 302; data from 1 patient is missing)

^b^After Bonferroni correction, the association for overall activity impairment remained statistically significant, with *P* < the Bonferroni correction-adjusted critical value of 0.0125

A similar pattern of results was observed when stratifying patients by VOC severity. Patients with more severe VOCs reported greater absenteeism, overall work productivity loss, and activity impairment in the 7 days preceding survey administration than patients with less severe VOCs (*P* < 0.05 for all) (Table 7; Panel b of Fig. 3).

**Table 7:** Work productivity according to VOC severity

WPAI:SHP domain^a^	Less severe VOCs					More severe VOCs					*P*^c^
	*N*^b^	Mean	SD	Median	IQR	*N*	Mean	SD	Median	IQR	
Absenteeism	77	21.12	23.91	16.67	0.00–37.50	41	39.59	33.96	30.77	10.00–62.50	0.003
Presenteeism	76	44.34	28.49	50.00	20.00–70.00	38	52.89	33.84	60.00	20.00–80.00	0.122
Overall work productivity loss	76	51.73	31.50	57.39	29.44–78.57	38	62.10	36.02	76.60	27.42–91.67	0.043
Overall activity impairment	188	48.78	27.77	50.00	30.00–70.00	114	60.35	29.36	70.00	40.00–80.00	0.001

*WPAI:SHP* Work Productivity and Activity Impairment: Specific Health Problem, *VOCs* vaso-occlusive crises, *SD* standard deviation, *IQR* inter-quartile range

^a^Higher WPAI:SHP scores indicate greater impairment. Absenteeism scores were calculated for patients who were employed at the time of the survey (*N* = 118). Presenteeism and overall work productivity scores were calculated for patients who were both employed and reported working in the past 7 (*N* = 114). Overall activity impairment scores calculated for all patients (*N* = 302; data from 1 patient is missing)

^b^More severe VOCs: ASCQ-Me Pain Episode Severity score ≥ 55 (½ SD worse than the benchmark score); less severe VOCs: ASCQ-Me Pain Episode Severity score < 55

^c^After Bonferroni correction, the association for absenteeism and overall activity impairment remained statistically significant, with *P* < the Bonferroni correction-adjusted critical value of 0.0125

## Discussion

The goal of this study was to explore the disease experience of patients with SCD, focusing primarily on better understanding the ways in which patients are impacted by the frequency and severity of VOCs. To our knowledge, this is one of the few studies designed to comprehensively investigate the impacts of both SCD and VOCs on multiple dimensions of patients’ lives. Data obtained from the cross-sectional online survey of adults with SCD provided evidence to demonstrate that patients experience impacts of SCD across many different aspects of their lives, including employment, education, and personal relationships. In addition, the frequency and severity of VOCs were associated with impacts on HRQoL and work productivity.

The frequency and severity of VOCs were related to multiple domains of HRQoL. In particular, patients with more frequent (or severe) VOCs reported greater impacts on areas known to be affected by SCD, as measured by the ASCQ-Me, than patients with less frequent (or severe) VOCs. Moreover, these patients experienced deficits in areas of emotional functioning, social functioning, and stiffness that were meaningfully lower than a benchmark SCD population. These findings extend past research that has linked the previous occurrence of VOCs to lower scores on general health, vitality, and bodily pain domains (as measured by the SF-36® Health Survey) [6] by demonstrating that HRQoL is impacted not only by any past experience of VOCs, but also by the frequency and severity of these experiences. The findings of the current study also highlight impacts that extend beyond physical functioning, illustrating the effects of VOCs on social and emotional functioning as well.

In addition to impacts on HRQoL, results demonstrate that the frequency of VOCs is associated with specific aspects of work productivity. The number of VOCs over a 12-month period was related to the amount of missed worktime in the 7 days preceding survey administration, suggesting that the impacts of VOCs on patients’ lives may extend beyond the end of the event itself. To the best of our knowledge, the WPAI has not been administered to patients with SCD in an observational setting. As such, the results obtained from this study cannot be compared to, or interpreted, in light of previous findings. However, findings can be compared to those reported in different disease areas, helping to better contextualize the current results. Specifically, the quantity of absenteeism reported by patients with the most frequent or severe VOCs is nearly double what has been reported by patients with non-malignant chronic pain (19.4%) [29], and by patients who have recently completed treatment for breast cancer (21–25%) [30], but slightly less than the absenteeism reported by patients who currently have breast cancer (56–61%) [30]. Absenteeism is calculated as the number of work hours missed, divided by the total number of hours a patient could have worked. Thus, an absenteeism score of 34.99 (the average score of patients with the most frequent VOCs) is generally equivalent to missing 14 h of a 40-h work week, while an absenteeism score of 39.59 (the average score of patients with the most severe VOCs) is generally equivalent to missing 16 h of a 40-h week. Put this way, the impacts of VOCs can be described more concretely, elucidating the ways in which employed patients with frequent or severe VOCs are impacted by SCD.

Given the degree of work impairment experienced by patients with SCD, and in particular by those with more frequent or severe VOCs, it is unsurprising that many patients also reported experiencing negative impacts on their overall employment status. Previous qualitative work has reported that patients with SCD find it difficult to manage their jobs. For example, the FDA’s Voice of the Patient report describes that patients with SCD experience difficulty keeping up with their work due to both absences from work and stress caused by various aspects of the disease [20]. Other qualitative research has documented patients’ descriptions of challenges related to finding and maintaining adequate employment; patients discussed difficulty keeping jobs or building job history, and having to leave jobs that were too physically demanding [10]. Our results extend these findings by helping to quantify the frequency with which patients experience such impacts, showing that such experiences are relatively widespread among patients with SCD. Similar to the impacts on employment, patients in our study also reported that their SCD had impacted their personal relationships and negatively impacted their education. Overall, the number of patients who experienced negative impacts on education was fewer than those who experienced negative impacts on employment. Patients with SCD often experience a difficult transition from pediatric to adult care [31], and may struggle to obtain consistent and effective care as young adults, thus potentially increasing the likelihood that SCD will negatively affect various facets of their adult lives. Indeed, approximately 75% of the patients in this study reported experiencing barriers to receiving care for their SCD, such as difficulty affording healthcare services, limited health insurance, discrimination by healthcare professionals, difficulty trusting healthcare professionals, and a lack of specialized treatment centers. Increasing access, options, and quality of SCD-related care may improve patients’ employment-related outcomes.

This study had some limitations. As with any information collected through patient report, recall bias could affect reports of events. Second, diagnosis of SCD was entirely self-reported. While recruitment of patients through collaboration with SCD-related organizations and advocacy groups in the absence of explicit physician confirmation has been reported elsewhere [32, 33], the trade-offs between relying on self-report (e.g., more expedient data collection, ability to recruit across a broad geographic region) and obtaining additional confirmation must be considered. Third, selection bias could affect the type of patients who participated in the survey; the survey could only be completed by individuals with internet access, and those who are unfamiliar or less comfortable using this type of technology may have been less likely to participate. Fourth, the study was designed to be cross-sectional, exploratory, and largely descriptive. As such, none of the relationships reported here can be interpreted as causal, nor can longitudinal relationships be inferred. However, the results of the study are informative in their own right and can provide a solid foundation for additional future research.

Balancing the aforementioned limitations, this study also had several particular strengths. First, the study sample was quite large, particularly for a rare disease. Second, evaluation of the study sample strongly suggests that it is generally representative of the larger SCD patient population. While more women than men completed the survey, scores obtained on the ASCQ-Me were nearly identical to those from an SCD benchmark population [23], and the distribution of patients across race, types of SCD, and across US geographic regions is similar to what has been reported in previous studies [34]. Third, the survey assessed a variety of different concepts related to the experience of patients with SCD, including both validated patient-reported outcome measures and items written specifically for this study. This approach allowed for a clearer assessment of the ways in which patients are impacted by the disease. For example, only relying on the WPAI to measure work-related outcomes would capture the experiences only of patients who were currently employed, failing to take into consideration the perspectives of unemployed patients, who comprised 61% of the total study sample. Rather, the WPAI was fielded in conjunction with a series of items regarding the lifetime experiences of all patients, regardless of current employment status, thus providing a more complete understanding of this particular outcome.

This study provides evidence to demonstrate a link between patient outcomes such as HRQoL and work impairment, and the frequency and severity of VOCs. The findings presented in this study provide a solid foundation for future research, which should aim to investigate a causal relationship between these factors. Additional research should also explore how health interventions or the alleviation of structural or environmental barriers to receiving healthcare may improve HRQoL and employment opportunities among patients with SCD.

In conclusion, this study provides a comprehensive description of the patient experience with SCD, with a specific emphasis on highlighting the ways in which VOC frequency and severity impact patients’ HRQoL and work productivity. This research provides evidence to suggest that VOCs may have broad and cumulative impact on aspects of life such as emotional and social functioning which may last beyond the end of the event itself.
